# Technoeconomic Analysis for Biodegradable and Recyclable Paper Coated with Synthetic Ionic PBAT for Packaging Application

**DOI:** 10.1021/acssuschemeng.4c04205

**Published:** 2024-08-06

**Authors:** Zahra Aayanifard, Christopher M. Saffron, Syeda Shamila Hamdani, Hazem M. Elkholy, Muhammad Rabnawaz

**Affiliations:** † School of Packaging, Michigan State University, 448 Wilson Road, East Lansing, Michigan 48824, United States; ‡ Department of Chemistry, Michigan State University, East Lansing, Michigan 48824, United States; § Department of Biosystems & Agricultural Engineering, Michigan State University, East Lansing, Michigan 48824, United States; ∥ Department of Chemical Engineering and Materials Science, Michigan State University, East Lansing, Michigan 48824, United States

**Keywords:** biodegradable, paper coating, PBAT, recyclable, technoeconomic analysis, production cost

## Abstract

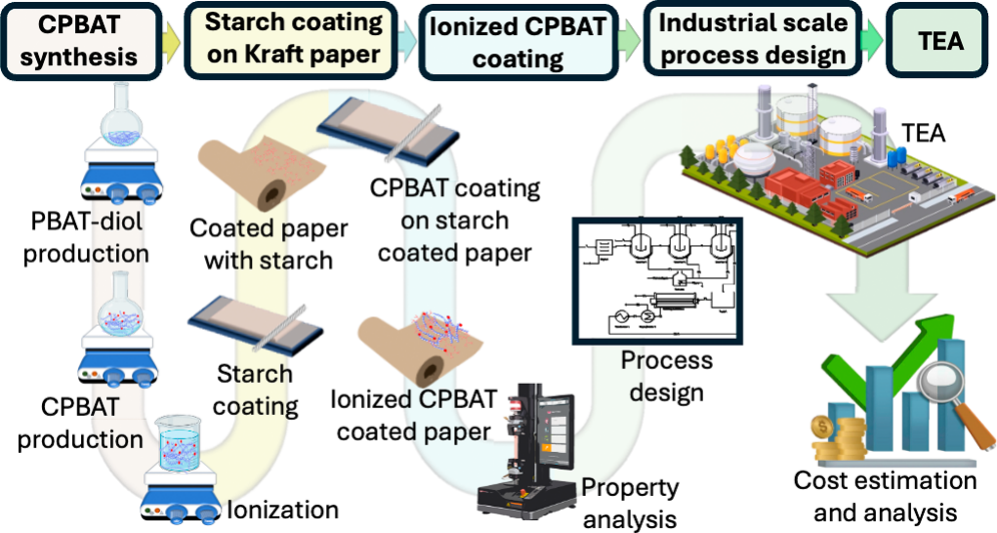

This study presents a technoeconomic analysis (TEA) for a novel ionic polybutylene adipate-*co*-terephthalate (PBAT), CPBAT, as a paper coating material, showcasing excellent water and oil resistance. This TEA determined total capital investment, operating costs, and minimum selling prices for a production capacity of 1,000 kg of CPBAT per day. The minimum selling prices of CPBAT coated on Kraft paper (CPBAT-K) and CPABT coated on starch-coated Kraft paper (CPBAT-S) are estimated to be $1.327/m^2^ and $1.864/m^2^, respectively. Additionally, the results of a sensitivity analysis show that the production of CPBAT-K and CPBAT-S is highly sensitive to the production capacity, raw material costs, energy efficiency of the coating process, reaction energy, and reaction yield. Recovery of the ionization solvent only marginally increases the selling prices of CPBAT-K and CPBAT-S, and hence, it is highly favorable. By increasing production capacity, lowering raw material costs, using energy-efficient coating machines, and partially recovering energy from reactions, the prices of CPBAT-K and CPBAT-S can be reduced to $0.588/m^2^ and $0.914/m^2^, respectively. Given that commercial polyethylene-coated paper prices range from $0.94/m^2^ to $1.850/m^2^, CPBAT-based coated papers with comparable mechanical and barrier properties along with biodegradability and recyclability are positioned as highly competitive and sustainable alternatives in the market.

## Abstract 2

A comprehensive technoeconomic analysis for the production of ionic PBAT-coated biodegradable and recyclable paper for packaging applications.

## 1 Introduction

Plastics are widely used for a large number of applications, especially in packaging; however, the microplastic issue has created significant problems. As a result, paper substrates are perceived as promising alternatives to plastics for single-use packaging applications. Paper offers many benefits; for example, it is inexpensive, flexible, biodegradable, and recyclable. In addition, due to strong hydrogen bonding between the fibrils, paper offers strength, providing desirable packaging properties like tensile strength, tear resistance, and flexibility.^1^

Despite the promising properties of paper substrates for packaging applications, serving as a barrier to liquids is a critical need. Water and oil penetrate through paper owing to hydrophilicity in the case of water and porosity in the cases of oil and water. Also, when water or oil penetrates through the paper, it weakens its performance and tensile properties because both liquids weaken the intermolecular forces between the fibers.

To address the above limitations, various treatments such as coating, lamination, or functionalization can be applied to enhance the mechanical and barrier properties of paper.^2^ Paraffin wax, per- and polyfluoroalkyl substances (PFAS),^3^ and low-density polyethylene (LDPE)^4^ are employed either as external sizing agents in paper coatings or as coatings/laminates to enhance the paper’s resistance to water and oil. Unfortunately, PFAS has been observed to leach from paper during repulsive processes, posing environmental risks. Furthermore, LDPE is nonbiodegradable and cannot be recycled through repulping processes.^5^ In addition to the external sizing agent, internal sizing agents are also used in offering paper to be water- and oil-resistant, such as the use of an alkyl ketene dimer (AKD) and octadecenylsuccinic anhydride (ODSA).

Our group has developed several PFAS- and plastic-free alternative paper coatings including starch-based,^5−7^ poly(vinyl alcohol) (PVOH)-based,^2,8^ coatings based on soybean oil,^9^ and chitosan-based.^6,10−15^ Despite their biodegradability, these polymers lack sufficient water resistance and also have weaker or no sealing capabilities required for effective packaging applications in commercial settings.^16^ We need polymeric coatings for paper to achieve a good water resistance and sealing performance. However, such coatings do not exist that offer excellent performance but also offer concurrent recyclable and biodegradable properties for the coated paper.

Polybutylene adipate-*co*-terephthalate (PBAT) is a commercial polymer, biodegradable, and suitable for packaging applications. Its full biodegradability, thermoplasticity, high flexibility, and easy processability are underlying reasons for its potential applicability as a coating material. However, the poor thermomechanical properties of PBAT can limit its mass application.^17,18^ To overcome these limitations, PBAT has been blended with starch and biofillers, including cellulose, lignin, and chitin.^19,20^ However, lignin has several limitations including thermal degradability, the tendency to agglomerate within host matrices, and variability in chemical structure depending on the feedstock source.^21^ Moreover, PLA/PBAT blends hold promise due to the combination of the mechanical strength of polylactic acid (PLA) and PBAT’s toughness.

In an attempt to create recyclable and biodegradable paper, our group recently synthesized an ionized PBAT (CPBAT) from commercially available PBAT followed by neutralization using ammonium hydroxide to generate a waterborne coating material.^22^ The obtained waterborne coating/emulsion was applied to Kraft paper and starch-coated paper. CPBAT has shown improved water and oil resistance, with prospects of recyclability and biodegradability. Herein, we report, for the first time, a detailed technoeconomic analysis of CPBAT production and its coating onto uncoated Kraft paper and starch-coated paper. No comprehensive TEA has been performed on PBAT or modified CPBAT-coated paper. This TEA determines the total capital investment for a production capacity of 1,000 kg of CPBAT per day and also estimates the minimum selling prices of CPBAT-K and CPBAT-S. Additionally, economic sensitivity analyses for the production of CPBAT-K and CPBAT-S are performed. Furthermore, the prices of CPBAT-K and CPBAT-S are compared to those of commercial Poly-coated papers.

## 2 Methods

**Figure 1:**
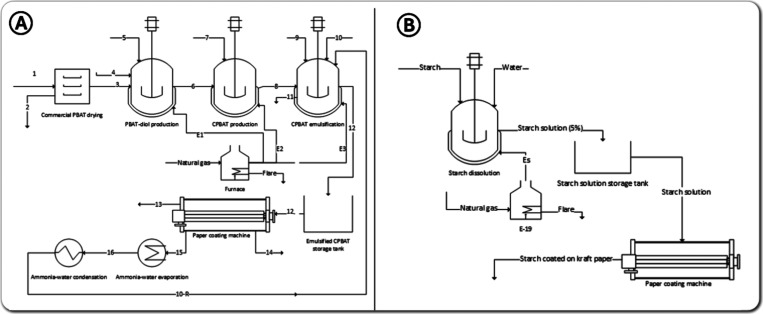
Process flow diagram for Kraft paper coated with CPBAT. (A) PFD of CPBAT production and Kraft paper coating and (B) PFD of 5% starch dissolution in water and coating Kraft paper.

A spreadsheet model was formulated starting from drying commercial PBAT (to remove moisture) to the final sale of CPBAT, CPBAT coated on Kraft paper (CPBAT-K), and CPBAT coated on starch-coated paper (CPBAT-S). A process flow diagram (PFD) (Figure 1) was drawn starting with drying PBAT in a rotary dryer followed by high-molecular-weight PBAT (10 molar ratio relative to the molecular weight of the repeating unit of PBAT) reacting with 1,4-butanediol (one molar ratio) to yield PBAT-diol of lower molecular weight. This reaction occurs in the presence of zinc acetate (0.5 wt %) as a catalyst in a continuous stirred tank reactor (CSTR) to yield low-molecular weight PBAT-diol within 6 h at 200 °C. Subsequently, this PBAT-diol was subjected to ring-opening addition reaction with *meso*-butane-1,2,3,4-tetracarboxylic dianhydride (MBTCA) in an equivalent molar ratio, resulting in the synthesis of CPBAT polymer, which occurs in a CSTR at 170 °C for 30 min.

To ionize CPBAT in water, CPBAT was mixed with ammonium hydroxide aqueous solution in a CSTR reactor for 45 min at 77 °C and transferred into a tank for storage. In the laboratory-scale experiments, the ionized CPBAT was initially coated on Kraft paper using a silicon spatula followed by drying at 130 °C for 30–40 min to produce CPBAT-K. Additionally, starch-coated paper was prepared by applying 5% starch solution onto uncoated Kraft paper using a multicoater machine (K303 multi coater) followed by air drying for 24 h. Subsequently, CPBAT was coated on the starch-coated paper to prepare CPBAT-S. It is assumed that in the industrial setting, press rolling coating machines are employed to coat ionized CPABT and starch solution onto Kraft paper. As part of this project, the coating machines are equipped with integrated dryers. Furthermore, it was assumed that the energy required for the reactions would be generated by burning natural gas in the furnaces, while other electricity demands would be supplied from mixed-grid electricity in the US.

To determine the minimum selling price, profitability analysis was conducted to initially estimate the annual cash flow and annual net and gross profit considering the internal rate of return of 0.1. The minimum selling price (MSP) of CPBAT production included all processes before the CPBAT emulsification and coating. While the cost and minimum selling price of CPBAT-K included the CPBAT coating on Kraft paper along with drying and recycling of the ionization solvent, the additional coating and drying for preparing starch-coated paper was incorporated into the costs and minimum selling price of CPBAT-S. To investigate the impact of various parameters on the costs and the minimum selling prices, a sensitivity analysis was performed to assess the impact of various parameters on the minimum selling prices of CPBAT-K and CPBAT-S. These parameters include the production capacity, reaction yield, energy required for the reactions, energy efficiency of the coating machines, internal rate of return, ionization solvent recovery rate, raw material costs, and price of PBAT.

The process was visualized in Visio software, while the equipment design utilized Aspen HYSYS and the literature.^23,24^ Equipment costs were determined through quotations from suppliers and insights from references.^23−26^ Chemical engineering plant cost indices (CEPCI) were employed to adjust equipment prices from previous years to the latest available index (2022). By combining these temporal cost adjustments with the six-tenths rule, the equipment costs were adjusted with the equipment scale.

To estimate the overall capital investments, the direct costs, including the instrumentation, piping, electrical systems, buildings, yard improvements, service facilities, and land, and indirect expenses, such as engineering, construction, legal fees, contractor charges, and contingency, were estimated based on purchased equipment costs. Direct operating costs were estimated by combining calculated process flow rates with the unitary costs of raw materials and utilities. Consequently, after estimating the raw material costs and utilities, fixed operating costs, including labor and benefits, supervision, laboratory expenses, consumables, insurance, taxes, depreciation, administrative overheads, and plant overheads, were derived from direct operating costs and capital investment values. For CPBAT production, it was determined that two operators, one supervisor, and one laboratory technician would be employed. For CPBAT-K production, two additional operators would be hired for the coating process and methanol recovery. In the CPBAT-K production process, an additional operator and laboratory technician were employed for the starch coating process. Additionally, assuming zero salvage value, depreciation was calculated based on a 10-year life by dividing the total capital investment by 10 (Table 1).

**Table 1:** Assumptions for TEA

**description**	**value**	**unit**	**reference**
plant capacity	1000	kg	
time horizon	10	years	
utility cost for mixed grid	0.1	USD kWh^–1^	(27)
utility cost for natural gas	0.016	USD kWh^–1^	(28)
PBAT	3.1416	USD kg^–1^	(20)
1,4-butanediol (BDO)	1.95	USD kg^–1^	(29)
zinc acetate	4	USD kg^–1^	(30)
MBCTA	6.39	USD kg^–1^	(31)
ammonium hydroxide	0.159	USD kg^–1^	(32)
starch	4.7	¢ kg^–1^	(33)
fresh water price	1.08	¢ kg^–1^	(34)
income tax	21	%	(35)
salvage value	0	%	
internal rate of return	0.1		

## 3 Results and Discussion

### 3.1 Fixed Capital Investment

**Figure 2:**
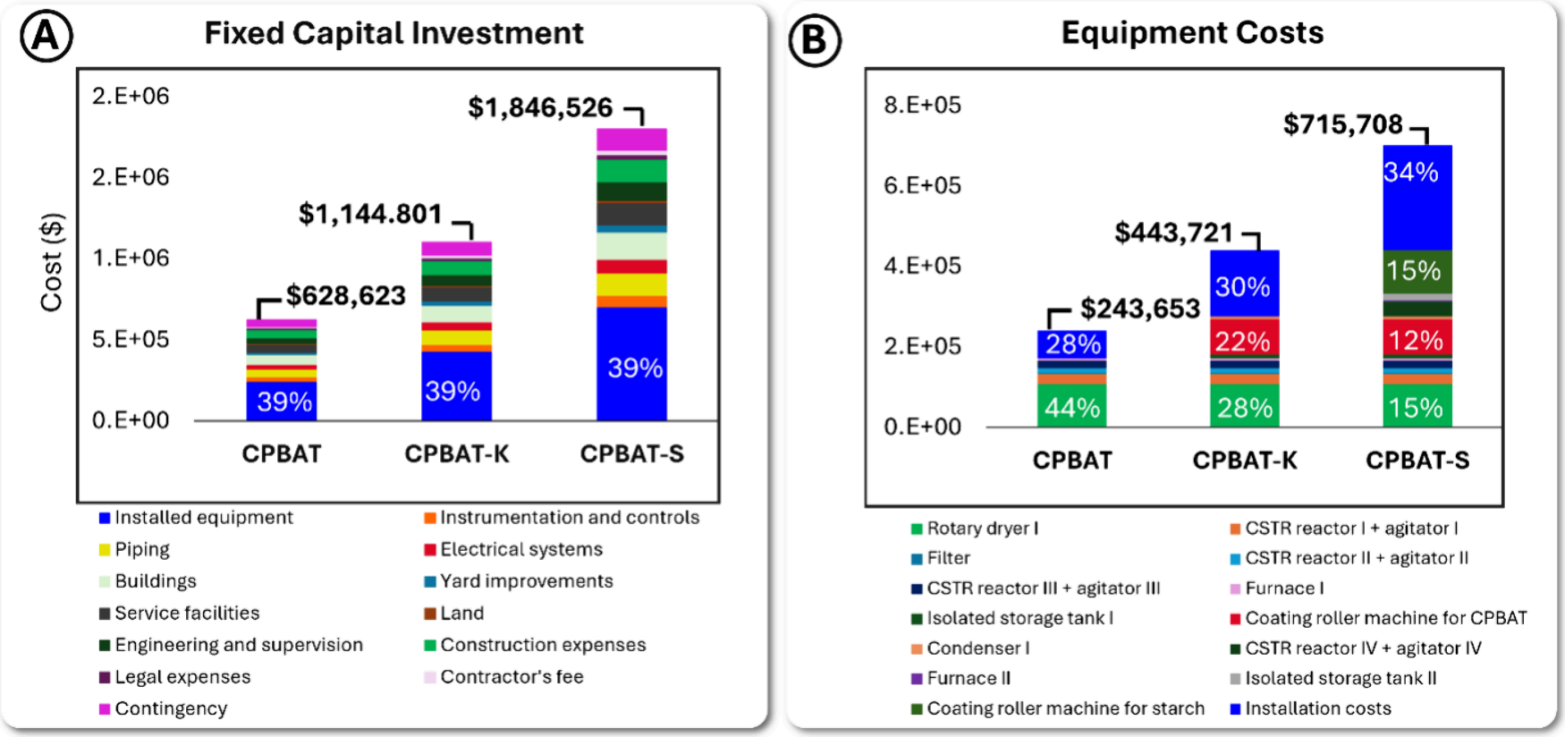
Breakdown of (A) fixed capital investment and (B) equipment costs for CPBAT, CPBAT-K, and CPBAT-S.

The stacked bar graph depicted in Figure 2A shows the breakdown of the costs comprising the fixed capital investment, highlighting that equipment costs are the major component of fixed capital investments. Since fixed capital investment is based on installed equipment costs, the summation of the equipment costs and installation costs contribute to 50% of non-normalized direct fixed capital investment and 39% after normalization to a total of 100%. Buildings (9%), piping (8%), and service facilities (8%) are the next largest contributors to direct costs, while other direct costs such as electrical systems (5%), instrumentation and controls (4%), yard improvement (2%), and land (1%) have lesser contributions. In terms of indirect costs, construction expenses and contingency (8%) are the two largest contributors, while engineering and supervision (6%), legal expenses (2%), and contractor’s fee (2%) make up the remaining contributors to the indirect cost portion of fixed capital investment. Additionally, the graph reveals that the fixed capital investments for the production of CPBAT-S are more than that of CPBAT-K.

In Figure 2B, the breakdown of equipment costs for CPBAT is exhibited, in which the emulsification and coating steps were not considered, with total equipment costs amounting to $174,620. However, the cost of one coating step in CPBAT-K production and two coating steps in CPABT-K increases the equipment costs to $279,340 and $432,759, respectively. This figure also reveals that while the rotary dryer for commercial PBAT drying constitutes the largest contributor to equipment costs of CPBAT and CPBAT-K, the sum of the two coating machine areas (total of 10 coating machines) is the largest contributor to CPBAT-S.

### 3.2 Operating Costs

**Figure 3:**
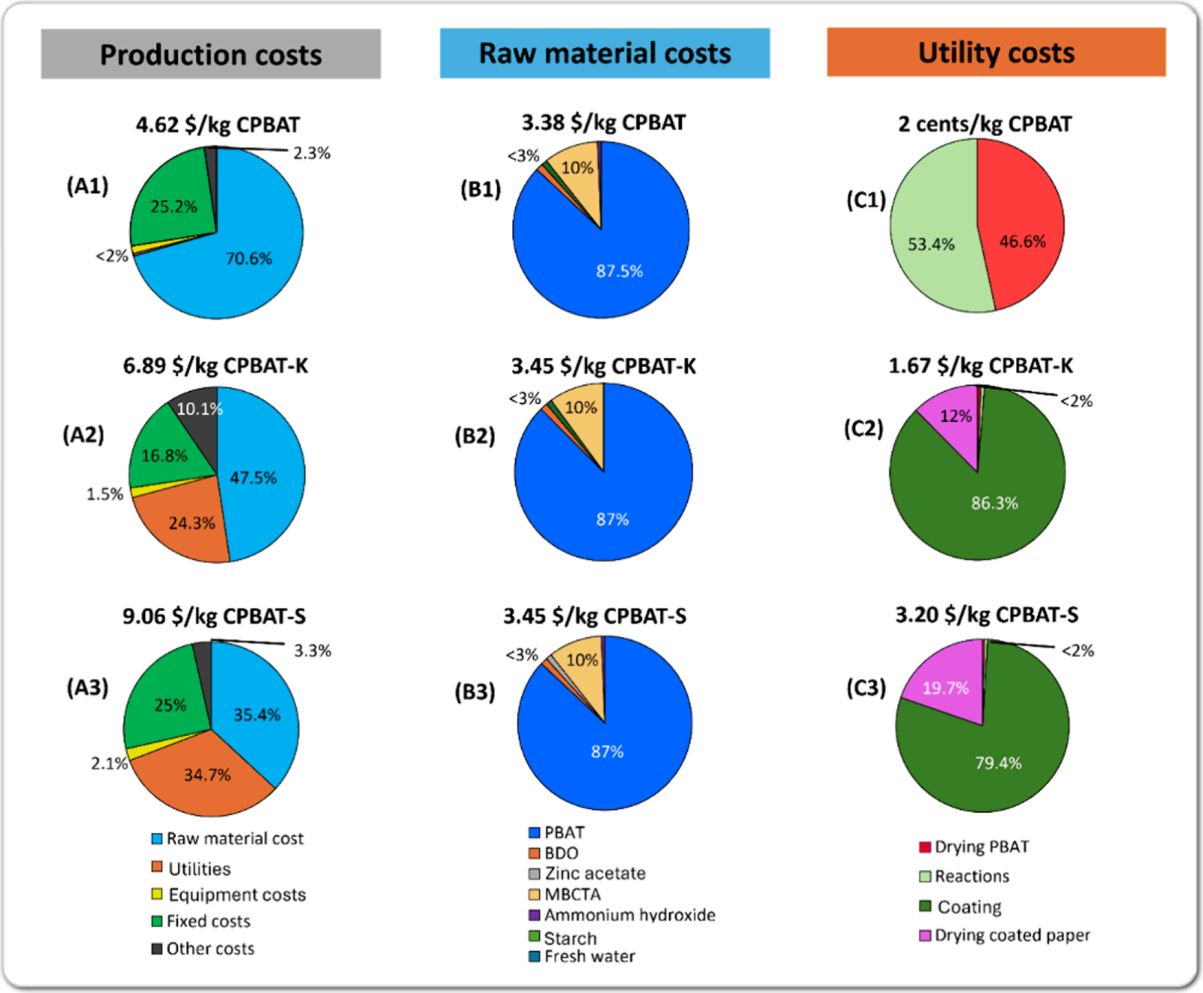
Production, raw material, and utility costs: utility costs (A1–3), production costs (B1–3), and raw material costs (C1–3). (A1–C1) Costs related to CPBAT production, (A2–C2) costs of CPBAT coated on Kraft paper, and (A3–C3) costs associated with CPBAT coated on starch-coated paper.

Figure 3 illustrates the production costs of CPBAT, CPBAT coated on Kraft paper (CPBAT-K), and starch-coated paper (CPBAT-S). CPBAT production involves drying and two production reactions, one of which is highly exothermic. Hence, the primary operating costs are raw materials, capital recovery charges, labor, and maintenance. However, for the production of CPBAT-K and CPBAT-S, high utility expenses arise due to energy consumption during coating and paper drying. Additionally, the equipment cost for CPBAT-S production exceeds that of CPBAT-K production owing to the five coating machines needed for the starch coating process and an extra reactor for starch dissolution in water. Figure 3 also shows the cost contributions of raw materials, revealing that PBAT is the key contributor to CPBAT, CPBAT-K, and CPBAT-S production followed by MBTCA as the second major contributor.

The contrast in utility costs between CPBAT production and CPBAT-K and CPBAT-S productions is remarkable. This difference primarily stems from energy-intensive coating processes. In CPBAT production, part of the utility costs is attributed to the endothermic reaction of CPBAT production and partly to the drying process of commercial PBAT. Conversely, in the CPBAT-K utility cost breakdown, the contribution of the reaction is marginal, with coating processes accounting for over 86% of the total. Similarly, in CPBAT-S, the majority of utility costs are linked to coating processes, while drying contributes approximately 20% of the total utility cost.

### 3.3 Profitability Analysis

**Figure 4:**
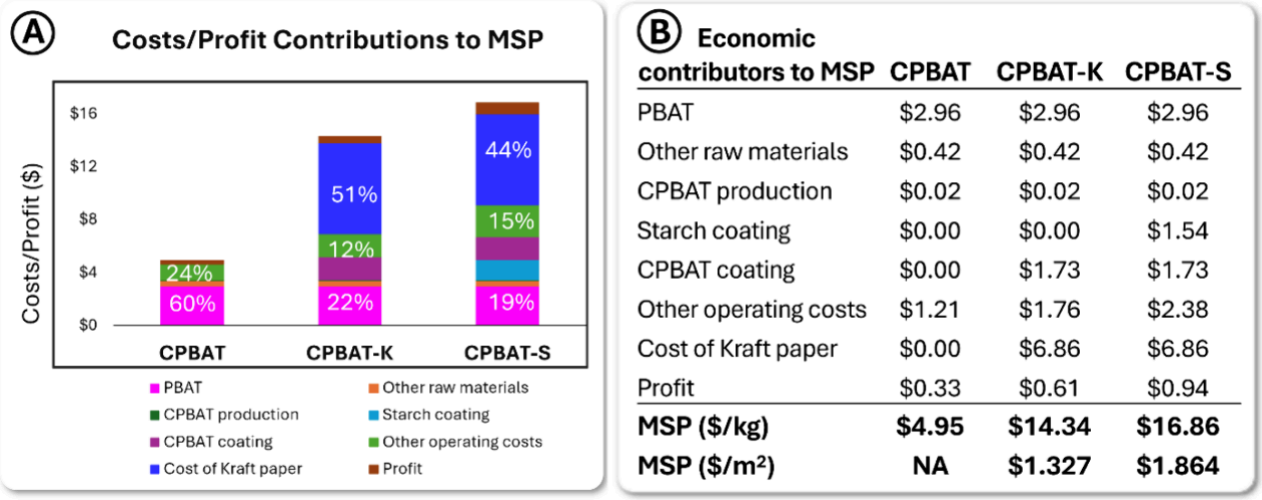
Itemization of costs and profits contributing to the minimum selling price (MSP) of CPBAT, CPBAT-K, and CPBAT-S. (A) Stacked bar chart for the components of MSP and (B) values for the costs and profit and MSP of CPBAT, CPBAT-K, and CPBAT-S.

Figure 4 shows the costs and benefits contributing to the minimum selling price (MSP) of CPBAT, CPBAT-K, and CPBAT-S, emphasizing that while PBAT is the major contributor to the minimum selling price of CPBAT, the price of Kraft paper is the key contributor to the cost of both papers. With an internal rate of return (IRR) of 10%, the MSP per kilogram of CPBAT-K is $14.41, while it amounts to $16.86 for CPBAT-S. Additionally, the MSP per square meter of CPBAT-K and CPBAT-S stands at $1.327 and $1.846, respectively. Considering the production cost of CPBAT at $4.62 (Figure 3) and the MSP of $4.95, this figure indicates that the main cost is associated with paper and coating costs. Optimizing the coating process by using a larger coating machine with higher production capacity and lower power consumption could reduce CPBAT-based coated paper prices.

### 3.4 Sensitivity Analysis

Once the utility costs, total operating costs, total capital investment, and minimum selling prices (MSPs) for CPBAT, CPBAT-K, and CPBAT-S were determined, changes in MSP were assessed across various parameters, such as production capacity, raw material costs, price of PBAT, CPBAT reaction yield, energy required for coating and reactions, internal rate of return (IRR), and rate of ionization solvent recovery. In the base case scenario, the production capacity was based on producing 1,000 kg of CPBAT per day, with a reaction yield of 100%, 40% recovery of the ionization solvent, and an IRR of 10%. Additionally, it was assumed that the energy of the exothermic reactions was not recovered and, hence, lost to the environment.

**Figure 5:**
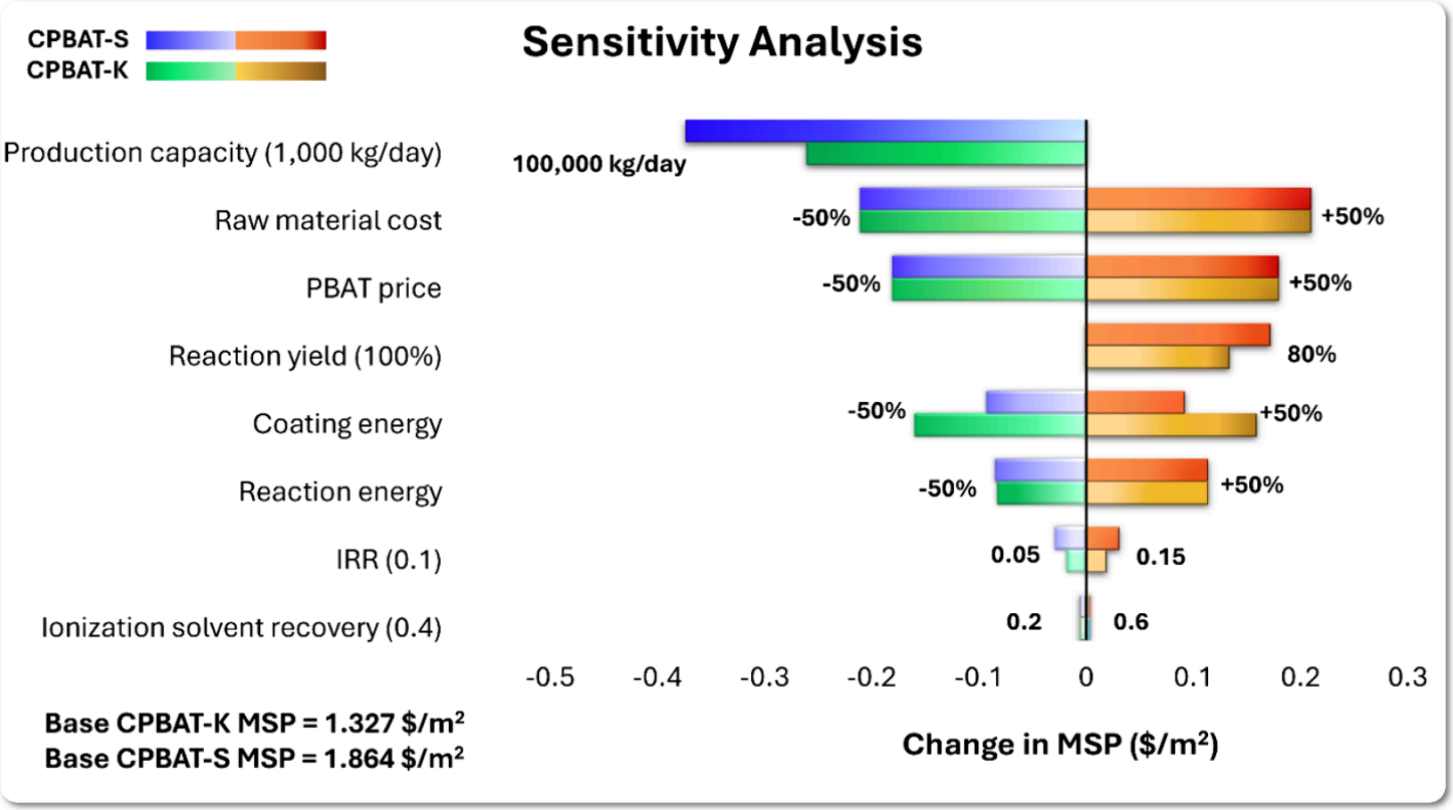
Sensitivity analysis: impact of changing various parameters on the MSPs of PBAT-K and PBAT-S, with the most sensitive parameter (production capacity) on the top and the least sensitive parameter at the bottom (ionization solvent recovery rate).

To analyze the sensitivity of the MSPs to the production capacity, the minimum selling prices for a larger plant with the capacity of producing 100,000 kg/day CPBAT were determined. For this purpose, the quantity of raw materials and required utility were scaled up, while the equipment costs were adjusted based on the six-tenths rule. The sensitivity analysis results, as depicted in Figure 5, revealed that the minimum selling prices of CPBAT-K and CPBAT-S are highly sensitive to the production capacity, and when expanding the plant size to 100,000 kg CPBAT production per day, the minimum MSPs drop by 20%. Studies also confirm that a larger production capacity in a chemical plant can be more cost-effective for manufacturers, provided that there is sufficient demand to justify the initial investment. This is because larger plants benefit from economies of scale, efficiency improvements, and better purchasing power, which lower the cost per unit produced.^36,37^

Additionally, due to the large contributions of material costs (specifically the price of PBAT) to the operating costs of CPBAT-K and CPBAT-S, the dependency of MSPs on the raw material costs and price of PBAT alone was examined by incorporating a 50% decrease/increase and recording the changes in the MSPs. The findings indicate that the minimum selling prices are highly influenced by raw material costs, particularly PBAT, which accounts for approximately 87% of the raw material costs. A 50% decrease in raw material costs could reduce the MSPs of CPBAT-K and CPBAT-S by 16 and 11%, respectively, while a corresponding increase could raise the MSPs by the same percentages. The changes in PBAT prices were closely associated with their contribution to overall raw material costs. Specifically, a 14% decrease and a 10% increase in MSPs were observed for CPBAT-K and CPBAT-S, respectively, reflecting changes in PBAT costs relative to total raw material costs.

The reaction yield was reduced by 80%, requiring extensive feed rate adjustments for raw material inputs. Nevertheless, as the reactor sizes were initially overestimated to accommodate lower yields, the overall capital investment remained unchanged. The 80% yield decrease led to 10 and 9% increases in MSPs for CPBAT-K and CPBAT-S, respectively, highlighting the need for optimizing reactions to attain higher product yields and thereby lower selling prices.

As outlined in Figure 3C2,C3, the energy requirement for the coating process was observed to have a considerable contribution to the utility costs of CPBAT-K and CPBAT-S. With a maximum coating rate of 7,200 m/day (width of 55 cm), five coating machines were required for each coating step, resulting in a high energy demand for coating. Hence, employing coating machines with greater capacity and enhanced energy efficiency can significantly influence costs and minimum selling prices (MSPs). Adjusting the energy demand to be 50% below and above the base case scenario resulted in a 12% decrease/increase in the MSP of CPBAT-K and a 5% change in the MSP of CPBAT-S.

As previously stated, the PBAT-diol production and CPBAT emulsification reactions are highly exothermic, generating recoverable energy for use in other process steps. However, a study on the depolymerization of polyethylene terephthalate (PET) suggested that despite the exothermic nature of the ester bond breakage reaction using a short diol, additional energy might be necessary to drive the reaction forward, as in the case of PBAT-diol production.^25^ To ensure consistent calculations, the energy demand for PBAT-diol production was determined through benchtop experiment scale-up. For instance, scaling up the energy requirement for breaking ester bonds in PBAT to produce lower-molecular-weight PBAT-diol required 66,577 MJ/day, compared to an estimate of around 99 MJ/day derived from large-scale energy requirements for breaking ester bonds in PET (as detailed in the Supporting Information). Although the scale-up energy calculation was notably higher due to the use of a glass laboratory apparatus with high energy loss, it is reasonable to consider a range of reaction energies between partial recovery of the produced energy in the exothermic reactions and partial energy demand calculated from the scale-up. As a result, modifying the reaction energy requirement from 50% energy recovery to 50% energy consumption led to a 6–9% decrease and increase in price for CPBAT-K and a 5–6% reduction and increase in the price of CPBAT-S.

Additionally, the internal rates of return (IRR) ranged between 5 and 15%, which impacted the minimum selling price by a profit margin due to shortened/extended periods of the expected return of the capital investment. Results exhibited price elevations of 1 and 2% for CPBAT-K and CPBAT-S, respectively, when the IRR increased to 15%, and similar price decreases when the IRR decreased to 5%. With a lower IRR, a longer time for return on investment is needed, a result of reduced profits owing to lower MSP.^38^

In this analysis, the sensitivity of minimum selling prices to the recovery of the ionization solvent was investigated. Elevating the recovery rate and reintroducing the recycled solvent into the emulsification step reduce raw material costs and harness energy from solvent condensation. However, this approach requires larger equipment with increased equipment costs, thereby raising capital investment. The results revealed that the dependence of MSPs on the recovery rate of the ionization solvent was marginal, with variations of less than 1% for both CPBAT-K and CPBAT-S.

### 3.5 Future Outlook

**Figure 6:**
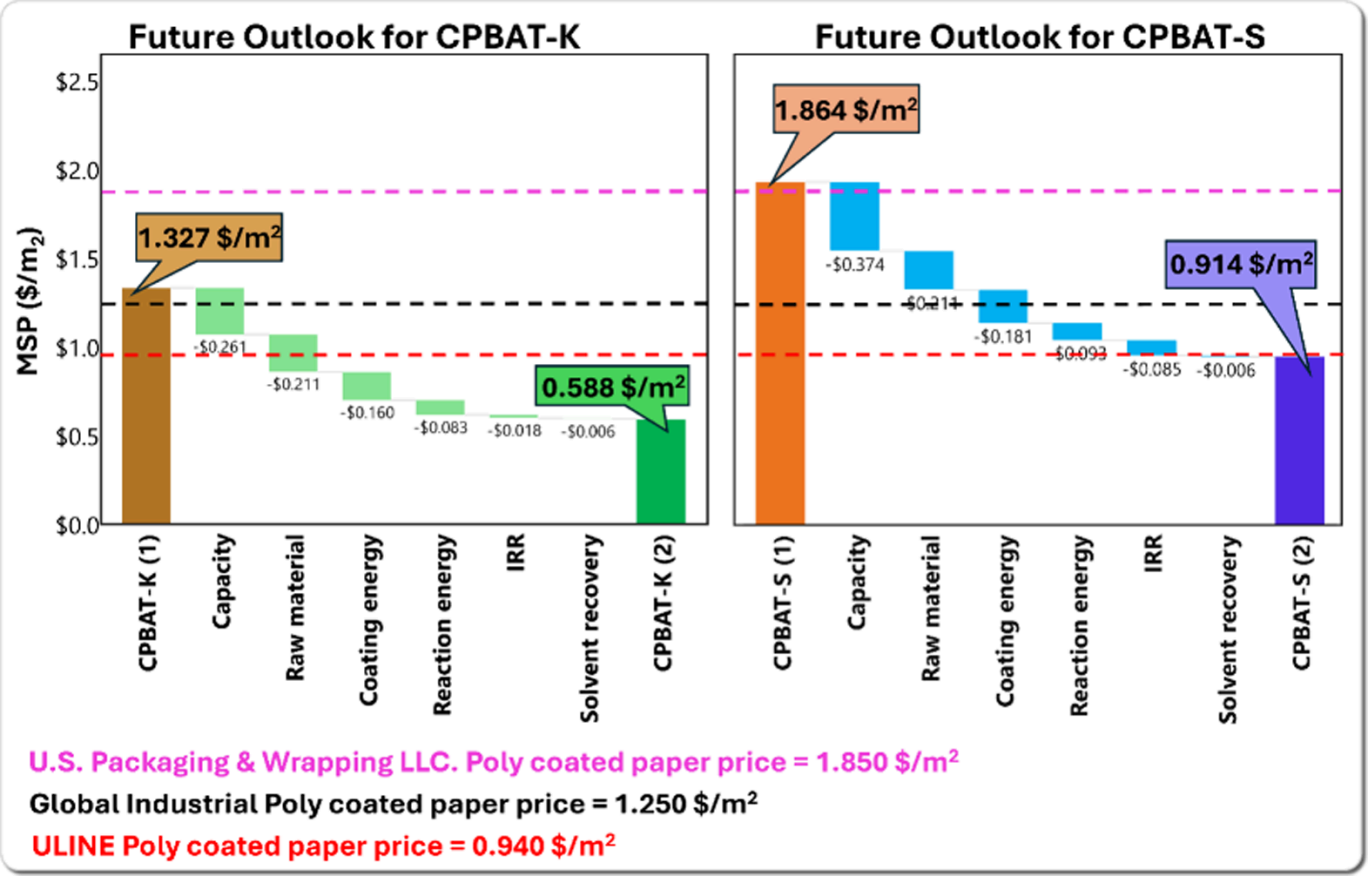
Future outlook for MSPs of CPBAT-K (on the left) and CPBAT-S (on the right): waterfall chart displaying cost-saving opportunities to reduce the minimum selling price of CPBAT-K and CPBAT-S.

Based on the results of the sensitivity analysis, a secondary scenario was formulated to explore the opportunities of lowering the minimum prices of CPBAT-K and CPBAT-S. In this scenario, the production capacity increased to 100,000 kg of CPBAT per day, with 50% lower-priced feedstock and 50% more energy-efficient coating machines. Additionally, insulated jacketed reactors with minimal energy loss were utilized, enabling 50% of the energy to be recovered and either redirected into other processes or sold at the same price as electricity generated from natural gas. With a reduced internal rate of return (IRR) of 5 and 20% recovery of the ionization solvent, the minimum prices dropped significantly to $0.588/m^2^ for CPBAT-K and $0.914/m^2^ for CPBAT-S, as shown in Figure 6. These prices were compared with commercially available Poly-coated papers from U.S. Packaging & Wrapping LLC., Global Industrial, and ULINE, priced at $1.85/m^2^, $1.25/m^2^, and $0.94/m^2^, respectively. This comparison reveals a feasible opportunity for CPBAT-K and CPBAT-S to achieve cost competitiveness while offering the added benefits of biodegradability and recyclability.

Although the initial cost of CPBAT-based papers is higher than that of ULINE Poly-coated paper, it is important to consider their recyclability and biodegradability, in contrast to the nonbiodegradable Poly-coated paper. Furthermore, our technoeconomic analysis is based on small-scale production of 1,000 kg per day, and scaling up the production capacity could significantly reduce costs. With the potential cost reduction by increasing the production scale and improving efficiency of the processes, CPBAT-K and CPBAT-S are promising alternatives for sustainable packaging solutions, filling a crucial gap in the market for environmentally friendly materials.

## 4 Conclusions

In this study, the economic feasibility of a novel ionic PBAT (CPBAT) as a paper coating material was investigated through a technoeconomic analysis (TEA). In a previous experimental study, CPBAT was initially applied to bare Kraft paper (CPBAT-K), demonstrating superior mechanical properties and good barrier properties. Additionally, when applied to starch-coated paper (CPBAT-S), it exhibited enhanced barrier properties compared with CPBAT-K. Importantly, both variants proved to be biodegradable and recyclable. This TEA determined the total capital investment, operating costs, and minimum selling prices for a production capacity of 1,000 kg of CPBAT per day. The total capital investments were estimated at approximately $1.14 M for CPBAT-K and $1.78 M for CPBAT-S. The estimation of operating costs highlighted significant contributions from raw material costs, particularly the cost of PBAT, and the energy requirements for the coating machines. Additionally, the estimated minimum selling prices for CPBAT-K and CPBAT-S are $1.327/m^2^ and $1.864/m^2^, respectively. A sensitivity analysis was conducted to provide insights into the dependence of minimum selling prices on various parameters, revealing the high sensitivity of MSPs to plant production capacity, raw material costs, energy efficiency of the coating process, energy required or released from reactions, and reaction yield. Furthermore, it is worth noting that the recovery of the ionization solvent and its reuse in the emulsification step only marginally increase the selling prices of CPBAT-K and CPBAT-S while saving energy and materials, making it highly recommended. In this scenario, the price of CPBAT-K falls within the range of commercial polyethylene-coated paper (PE paper/Poly-coated paper), priced from $0.94 to $1.85, while CPBAT-S is 7–96% more expensive. By increasing production capacity, lowering raw material costs, using more energy-efficient coating machines, and partially recovering energy from reactions, the MSPs can be reduced to $0.588/m^2^ for CPBAT-K and $0.914/m^2^ for CPBAT-S. In conclusion, with comparable mechanical and barrier properties to PE paper and the added benefits of biodegradability and recyclability, CPBAT-based papers offer a cost-competitive and sustainable alternative to current coated paper packaging.

## Supporting Information Available

Highlights; abbreviations; overview of the process; process flow diagram for Kraft paper coated with CPBAT, PFD of CPBAT production and Kraft paper coating, and PFD of 5% starch dissolution in water and coating Kraft paper; label definitions for the process flow diagram; stream table; dryer (A) specification; parameters for designing the dryer; DRYPLUS model DP160 desiccant wheel dryer for PBAT drying; specification of the industrial dryer; hot plate specifications; schematics of the hot plate and energy inputs and output; summary of reactions and energy requirements; specifications of the coating machine; Xiamen Simy customizable coating line solvent coater machine with an oven; data for CPBAT-coated unbleached Kraft paper from ULINE (75 lb); data for starch coated on unbleached Kraft paper from ULINE (75 lb), water–ammonia evaporation modeling using Aspen HYSYS; summary of equipment costs and sizing for CPBAT, CPABT-K, and CPBAT-S production; water–ammonia condensation modeling using Aspen HYSYS (20% loss) (PDF)

## Notes

H.M.E. is on leave from the Department of Chemistry, Faculty of Science, Tanta University, Tanta, 31527, Egypt.

## References

[ref1] Aguado R.; Murtinho D.; Valente A. J. M. A broad overview on innovative functionalized paper solutions. Nord. Pulp Pap. Res. J. 2019, 34 (4), 395–416. 10.1515/npprj-2019-0036.

[ref2] Hamdani S. S.; Li Z.; Sirinakbumrung N.; Rabnawaz M. Zein and PVOH-Based Bilayer Approach for Plastic-Free, Repulpable and Biodegradable Oil- And Water-Resistant Paper as a Replacement for Single-Use Plastics. Ind. Eng. Chem. Res. 2020, 59 (40), 17856–17866. 10.1021/acs.iecr.0c02967.

[ref3] Trier X.; Taxvig C.; Rosenmai A. K.; Pedersen G. A. Pfas in Paper and Board for food contact. 2018. Nordic Council of Ministers. 10.6027/TN2017–573.

[ref4] Moynihan R. H.; Baird D. G.; Ramanathan R. Additional observations on the surface melt fracture behavior of linear low-density polyethylene. J. Nonnewton. Fluid Mech. 1990, 36 (C), 255–263. 10.1016/0377-0257(90)85012-N.

[ref5] Kansal D.; Hamdani S. S.; Ping R.; Rabnawaz M. Starch and Zein Biopolymers as a Sustainable Replacement for PFAS, Silicone Oil, and Plastic-Coated Paper. Ind. Eng. Chem. Res. 2020, 59 (26), 12075–12084. 10.1021/acs.iecr.0c01291.

[ref6] Nair A.; Kansal D.; Khan A.; Rabnawaz M. New alternatives to single-use plastics: Starch and chitosan- graft -polydimethylsiloxane-coated paper for water- and oil-resistant applications. Nano Sel. 2022, 3 (2), 459–470. 10.1002/nano.202100107.

[ref7] Hamdani S. S.; Li Z.; Rolland E.; Mohiuddin M.; Rabnawaz M. Barrier and mechanical properties of biodegradable paper bilayer-coated with plasticized starch and zein. J. Appl. Polym. Sci. 2023, 140 (8), 1–15. 10.1002/app.53440.

[ref8] Hamdani S. S.; Li Z.; Ruoqi P.; Rollend E.; Rabnawaz M. Oxygen and water vapor barrier properties of polyvinyl alcohol and zein bilayer-coated paper. J. Appl. Polym. Sci. 2022, 139 (7), 51707 10.1002/app.51707.

[ref9] Kumar V.; Khan A.; Rabnawaz M. A plant oil-based eco-friendly approach for paper coatings and their packaging applications. Prog. Org. Coat. 2023, 176, 107386 10.1016/j.porgcoat.2022.107386.

[ref10] Kansal D.; Rabnawaz M. Fabrication of oil- and water-resistant paper without creating microplastics on disposal. J. Appl. Polym. Sci. 2021, 138 (3), 49692 10.1002/app.49692.

[ref11] Li Z.; Rabnawaz M. Oil- And Water-Resistant Coatings for Porous Cellulosic Substrates. ACS Appl. Polym. Mater. 2019, 1 (1), 103–111. 10.1021/acsapm.8b00106.

[ref12] Li Z.; Rabnawaz M.; Khan B. Response Surface Methodology Design for Biobased and Sustainable Coatings for Water- And Oil-Resistant Paper. ACS Appl. Polym. Mater. 2020, 2 (3), 1378–1387. 10.1021/acsapm.9b01238.

[ref13] Li Z.; et al. A closed-loop and sustainable approach for the fabrication of plastic-free oil- And water-resistant paper products. Green Chem. 2019, 21 (20), 5691–5700. 10.1039/C9GC01865D.

[ref14] Kansal D.; Hamdani S. S.; Ping R.; Sirinakbumrung N.; Rabnawaz M. Food-Safe Chitosan-Zein Dual-Layer Coating for Water- And Oil-Repellent Paper Substrates. ACS Sustain. Chem. Eng. 2020, 8 (17), 6887–6897. 10.1021/acssuschemeng.0c02216.

[ref15] Nair A.; Kansal D.; Khan A.; Rabnawaz M. Oil- and water-resistant paper substrate using blends of chitosan-graft-polydimethylsiloxane and poly(vinyl alcohol). J. Appl. Polym. Sci. 2021, 138 (21), 1–12. 10.1002/app.50494.

[ref16] Chen L.; Li P.; Guan J.; Xu C.; Xu C. A.; Yang Z. Castor oil-based paper packaging coating with water resistance and degradability obtained by thiol-ene click reaction. J. Appl. Polym. Sci. 2024, 141, 1–13. 10.1002/app.55269.

[ref17] Debeli D. K.; Wu L.; Huang F. PBAT-based biodegradable nanocomposite coating films with ultra-high oxygen barrier and balanced mechanical properties. Polym. Degrad. Stab. 2023, 216 (July), 110489 10.1016/j.polymdegradstab.2023.110489.

[ref18] Shankar S.; Rhim J. W. Effects of poly(butylene adipate-co-terephthalate) coating on the water resistant, mechanical, and antibacterial properties of Kraft paper. Prog. Org. Coatings 2018, 123 (April), 153–159. 10.1016/j.porgcoat.2018.07.002.

[ref19] Xiong S. J.; et al. A strong, tough and cost-effective biodegradable PBAT/lignin composite film via intrinsic multiple noncovalent interactions. Green Chem. 2023, 25 (8), 3175–3186. 10.1039/D2GC04929E.

[ref20] Xiong S. J.; et al. Economically Competitive Biodegradable PBAT/Lignin Composites: Effect of Lignin Methylation and Compatibilizer. ACS Sustain. Chem. Eng. 2020, 8 (13), 5338–5346. 10.1021/acssuschemeng.0c00789.

[ref21] Shorey R.; Mekonnen T. H. Sustainable paper coating with enhanced barrier properties based on esterified lignin and PBAT blend. Int. J. Biol. Macromol. 2022, 209 (PA), 472–484. 10.1016/j.ijbiomac.2022.04.037. 35413316

[ref22] Elkholy H. M. Design of carboxylic acid-functionalized poly(butylene adipate-co-terephthalate) for recyclable and biodegradable zero-waste paper packaging (in review). 2024; pp 1–40.

[ref23] Peters M. S.; Timmerhaus K. D. Plant design and economics for chemical engineers. McGraw-Hill: New York, 1968.

[ref24] Singh A.; et al. Techno-economic, life-cycle, and socioeconomic impact analysis of enzymatic recycling of poly(ethylene terephthalate). Joule 2021, 5, 2479–2503. 10.1016/j.joule.2021.06.015.

[ref25] Huang P. “Peng Huang, Ashiq Ahamed, Ruitao Sun, Guilhem X. De Hoe, Joe Pitcher, Alan Mushing, Fernando Lourenc ¸ o, and Michael P. Shaver *,”. 2023. 10.1021/acssuschemeng.3c04047 PMC10598876 37886038

[ref26] Uekert T.; et al. Technical, Economic, and Environmental Comparison of Closed- Loop Recycling Technologies for Common Plastics. ACS Sustain. Chem. Sci. Eng. 2023, 11 (3), 965–978. 10.1021/acssuschemeng.2c05497.

[ref27] “Illinois Electricity Rates,” 2024. https://www.energybot.com/electricity-rates/illinois/.

[ref28] “US Energy Information Association,” 2024. https://www.energybot.com/electricity-rates/illinois/.

[ref29] “Track Butanediol Price Trend in Top 10 Leading Countries Worldwide,” 2024, https://www.chemanalyst.com/Pricing-data/butanediol-54.

[ref30] “Zinc Acetate 99.9% Granular (Powder) ACS Grade,” 2023, https://www.laballey.com/products/zinc-acetate-granular-acs?variant=42654366531739&currency=USD&utm_medium=product_sync&utm_source=google&utm_content=sag_organic&utm_campaign= sag_organic&gad_source=1&gclid=Cj0KCQiAr8eqBhD3ARIsAIe-buNNULfcuh-3UyZWP7z0OuI6k.

[ref31] “meso-Butane-1,2,3,4-tetracarboxylic Acid, 98% purity,” 2023. https://www.aladdinsci.com/m158399.html?gclid=Cj0KCQiAj_CrBhD-ARIsAIiMxT8YsGQ4uJkxaQpTUfP030xeN4mjaBhUi7y3nFLga-mUghvMJbUAuQcaArkbEALw_wcB.

[ref32] “Ammonium Hydroxide Prices Current and Forecast,” 2019. https://www.intratec.us/chemical-markets/ammonium-hydroxide-price.

[ref33] “Industrial Grade Starch Powder Manufacturer Price,” 2024. https://www.alibaba.com/product-detail/Industrial-Grade-Corn-Starch-Powder-Manufacturer_1600696058510.html?s=p.

[ref34] Jadidi Y.; Frost H.; Das S.; Liao W.; Draths K.; Saffron C. M. Comparative Life Cycle Assessment and Technoeconomic Analysis of Biomass-Derived Shikimic Acid Production. ACS Sustain. Chem. Eng. 2023, 11 (33), 12218–12229. 10.1021/acssuschemeng.2c06681.

[ref35] “Corporate Tax Rates 2023,” 2023, https://www2.deloitte.com/content/dam/Deloitte/global/Documents/Tax/dttl-tax-corporate-tax-rates-2019-2023.pdf.

[ref36] Hsu C. I.; Li H. C. An integrated plant capacity and production planning model for high-tech manufacturing firms with economies of scale. Int. J. Prod. Econ. 2009, 118 (2), 486–500. 10.1016/j.ijpe.2008.09.015.

[ref37] Radatz H.; Kühne K.; Bramsiepe C.; Schembecker G. Comparison of capacity expansion strategies for chemical production plants. Chem. Eng. Res. Des. 2019, 143, 56–78. 10.1016/j.cherd.2018.12.018.

[ref38] Jurčević J.; Pavić I.; Čović N.; Dolinar D.; Zoričić D. Estimation of Internal Rate of Return for Battery Storage Systems with Parallel Revenue Streams: Cycle-Cost vs. Multi-Objective Optimisation Approach. Energies 2022, 15 (16), 5859 10.3390/en15165859.

