# Supplementary material for: Technoeconomic Analysis for Biodegradable and Recyclable Paper Coated with Synthetic Ionic PBAT for Packaging Application
Source: ACS Sustain Chem Eng. 2024 Aug 6;12(33):12576–83. doi: 10.1021/acssuschemeng.4c04205 (PMC11337150; doi:10.1021/acssuschemeng.4c04205)
Supplement: Supplementary file 1 — sc4c04205_si_001.pdf [file sc4c04205_si_001.pdf]

Supporting Information

Technoeconomic Analysis for Biodegradable and Recyclable Paper Coated with Synthetic Ionic PBAT For Packaging Application

Zahra Aayanifard[1,2#], Christopher M. Saffron[3,4#], Syeda Shamila Hamdani[1], Hazem M. Elkholy[1], Muhammad Rabnawaz[1,2]*

[1]School of Packaging, Michigan State University, 448 Wilson Road, East Lansing, Michigan, 48824-1223, United States

[2]Department of Chemistry, Michigan State University, East Lansing, Michigan 48824, United States

[3]Department of Biosystems & Agricultural Engineering, Michigan State University, East Lansing, Michigan 48824, United States

[4]Department of Chemical Engineering and Material Science, Michigan State University, East Lansing, Michigan 48824, United States

*Corresponding author: rabnawaz@msu.edu

[#]Authors with equal contribution

Number of pages: 22
Number of figures: 5
Number of tables: 14

Supporting Information

Highlights

- The total capital investment for a production capacity of 1000 kg of CPBAT per day is \$1,144,801 for CPBAT-K and \$1,777,453 for CPBAT-S.
- The minimum selling prices of CPBAT-K and CPBAT-S are estimated to be \$1.327/m^2 and \$1.864/m^2, respectively.
- The production of CPBAT-K and CPBAT-S is highly sensitive to the production capacity, energy efficiency of the coating machines, and energy required or released for/from the reactions.
- Optimizing production can reduce CPBAT-K and CPBAT-S prices to \$0.588/m^2 and \$0.920/m^2, making them competitive with commercial polyethylene coated papers priced between \$0.94/m^2 and \$1.850/m^2, with advantage of biodegradability and recyclability.
- Recovery of the ionization solvent recovery marginally increases the minimum selling prices of CPBAT-K and CPBAT-S, therefore it is highly suggested.

Abbreviations

**PBAT**	Polybutylene adipate-co-terephthalate
**CPBAT**	Carboxylic acid-modified polybutylene adipate-co-terephthalate
**CPBAT-K**	Carboxylic acid-modified polybutylene adipate-co-terephthalate coated on Kraft paper
**CPBAT-S**	Carboxylic acid-modified polybutylene adipate-co-terephthalate coated on starch coated paper
**TEA**	Technoeconomic analysis
**MSP**	Minimum selling price
**PFAS**	Per- and polyfluoroalkyl substances
**IRR**	Internal rate of return
**PE**	Polyethylene
**Poly**	Polyethylene
**PLA**	Polylactic acid
**ODSA**	Octadecenylsuccinic anhydride

Supporting Information

**PVOH**	Polyvinyl alcohol
**AKD**	Alkyl ketene dimer
**PET**	Polyethylene terephthalate
**BDO**	Butanediol
**MBT**	
**CA**	Meso-butane-1,2,3,4-tetracarboxylic dianhydride
**PFD**	Process flow diagram
**CSTR**	Continuous stirred tank reactor
**CEPC**	
**I**	Chemical engineering plant cost indices

Supporting Information

This section details the calculation methods used to perform the technoeconomic analysis (TEA).

This section details the calculation methods used to perform the technoeconomic analysis (TEA).

Overview of the Process

A Process flow diagram is in ***Figure S1*** that shows the production starts with drying PBAT in a rotary dryer, then the dried PBAT is reacted with 1,4-butanediol in the presence of zinc acetate as a catalyst in a continuous stirred tank reactor (CSTR), PBAT-diol production reactor, to yield smaller polymer chains of PBAT-diol within 6 hours at 200 °C. PBAT-diol is then allowed to react with meso-butane-1,2,3,4-tetracarboxylic dianhydride (MBTCA) in another CSTR, CPBAT production reactor, at 170 °C for 30 min to produce CPBAT. To emulsify CPBAT in water, it is mixed with aqueous ammonium hydroxide solution in a CSTR, CPBAT emulsification reactor, for 45 min at 77 °C and transferred into tank I for storage. The energy for reactions is generated by the combustion of natural gas in a furnace. The cost and minimum selling price (MSP) of production of CPBAT include drying PBAT and the first two reaction steps. For finding the cost and minimum selling price of kraft paper coated with CPBAT, the coating step along with drying is included. The coating machine on the left side is used to coat CPBAT on kraft paper or starch coated kraft paper and is a press coating machine with the drying unit that is integrated into it. To analyze the impact of the coating step alone, the drying step is designed separately.

Supporting Information

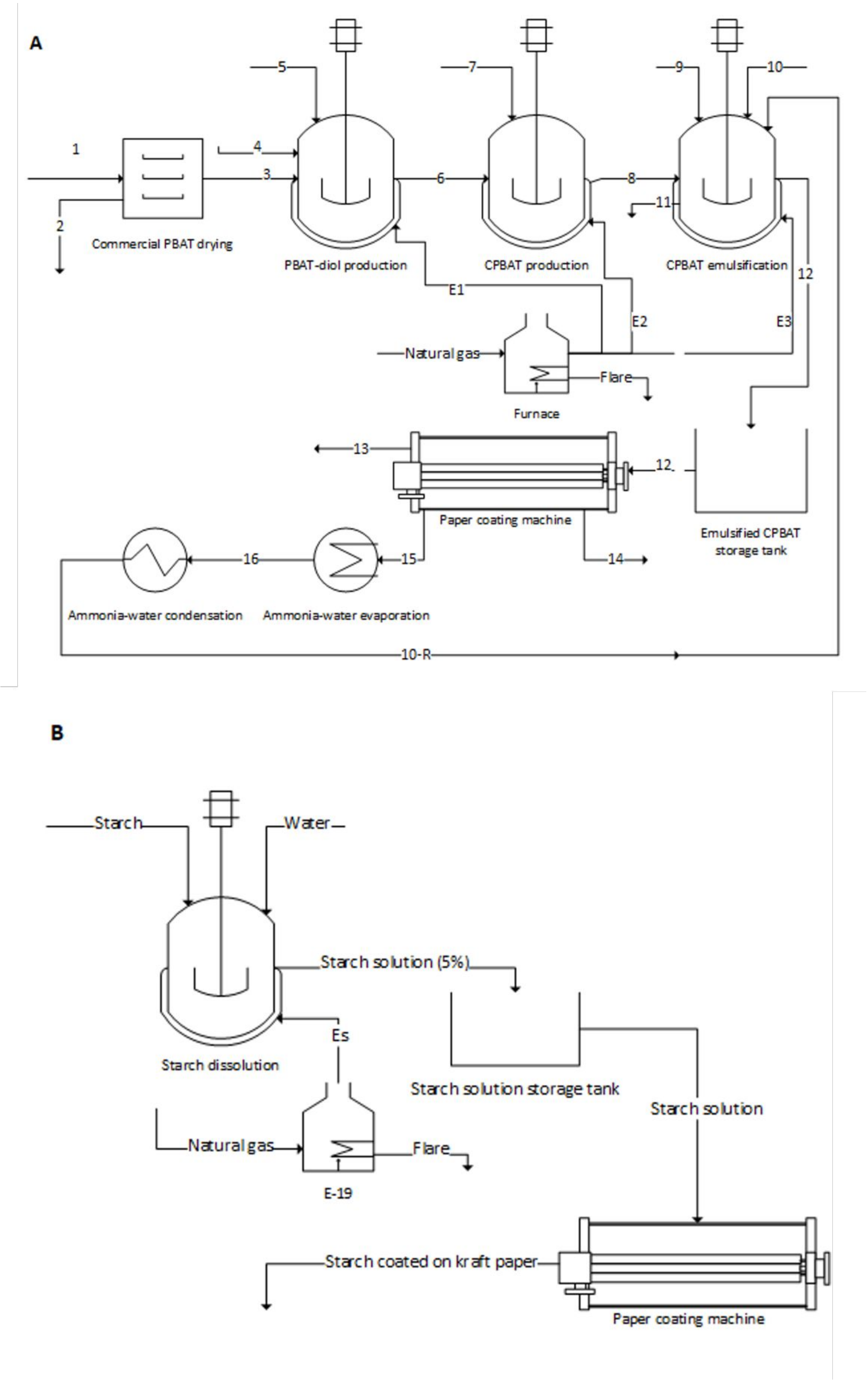

Figure S1. *Process Flow Diagram for kraft paper coated with CPBAT. A) PFD of CPBAT production and kraft paper coating; B) PFD of 5% starch dissolution in water and coating kraft paper*

Supporting Information

Evaporated ionization/emulsification solvent is condensed, in an ionization solvent condenser, and recycled back into the CPBAT emulsification reactor.

Table S1. *Label Definitions for the Process Flow Diagram*

Equipment	Process
**CPBAT Production and Coating Process (A)**	
**First dryer**	Drying commercial PBAT
**First reactor**	PBAT-diol production reaction
**Second reactor**	CPBAT production reaction
**Third reactor**	CPBAT emulsification reaction
**Furnace**	Natural gas combustion
**Tank**	CPBAT emulsion storage
**Coating machine**	Coating CPBAT emulsion
**Second dryer/boiler (built in coating machine)**	Drying CPBAT coated paper
**Condenser**	Condensation of water-ammonium mixture
**Starch Coating Process (B)**	
**Reactor**	Starch dissolution
**Furnace**	Natural gas combustion
**Tank**	Starch solution storage
**Coating machine with built-in dryer**	Drying starch coated paper

Below is the stream table according to ***Error! Reference source not found.***.

Supporting Information

Table S2. *Stream table*

	PBAT	*BDO*	*Zn(OAc)₂*	*PBAT-Diol*	*MBTCA*	*CPBAT*	*NH₄-OH*	*Water*	*CPBAT emulsion*	*Starch*	*P*	*T*
**Stream #**	*kg/day*	*kg/day*	*kg/day*	*kg/day*	*kg/day*	*kg/day*	*kg/day*	*kg/day*	*kg/day*	*kg/day*	*kPa*	*°C*
**1**	924.1	--	--	--	--	--	--	18.9	--	--	101	25
**2**	--	--	--	--	--	--	--	18.9	--	--	101	70
**3**	924.1	--	--	--	--	--	--	--	--	--	101	70
**4**	--	22.7	--	--	--	--	--	--	--	--	101	25
**5**	--	--	9.2	--	--	--	--	--	--	--	101	25
**6**	--	--	--	946.8	--	--	--	--	--	--	101	200
**7**	--	--	--	--	53.2	--	--	--	--	--	101	25
**8**	--	--	--	--	--	1,000		--	--	--	101	25
**9**	--	--	--	--	--	--	692.3	--	--	--	101	25
**10**	--	--	--	--	--	--	--	1,923.1	--	--	101	25
**11**	--	--	9.2	--	--	--	--	--	--	--		
**12**	--	--	--	--	--	--	--	--	3,615.4	--	101	77
**13**	--	--	--	--	--	1,000	--	--	--	--	101	77
**14**	--	--	--	--	--	--	138.5	384.6		--	101	77
**15**	--	--	--	--	--	--	553.8	1,538.5		--		
**16**	--	--	--	--	--	--	553.8	1,538.5	--	--	101	77
**10-R**	--	--	--	--	--	--	553.8	1,538.5	--	--	101	160

Drying wet PBAT

Commercial PBAT usually has 1-2% water. Herein, it is assumed that the commercial PBAT has 2% water. The energy for drying PBAT goes both for evaporating water and preheating PBAT to the reaction temperature in reactor I. Ideally, moisture content was reduced to 0%.

The feedstock data have been summarized in ***Table S3***.

Table S3. *Dryer (A) specification*

**Parameter**	**Value**	**Unit**
**Input to dryer**	Wet PBAT	
**Wet moisture % (X_i)**	2	%
**Dried moisture % (Xo)**	0	%
**Air moisture in (Y_i)**	1.5	%
**Air moisture out (Yo)**	3.2	%
**$C_{p,w}$**	4.19	kJ/kg °C
**ΔH_w^{vap} (at 120 °C)**	2.05	MJ/kg
**Mass flow rate of water (L_s)**	18.86	kg/day
**Mass flow rate of gas (G_s)**	1,087.60	kg/day
**$C_{p,PBAT}$**	1.5	kJ/kg °C

**h_{water} (at 120 °C)**	2.05	MJ/kg
**Energy requirement for preheating water**	7.50	MJ/day
**Energy requirement for water evaporation**	38.69	MJ/day
**h_{PBAT}**	1.50	KJ/kg °C
**T_{rxn}**	200	°C
**Energy requirement for preheating PBAT**	242.58	MJ/day
**Dryer efficiency**	0.5	NA
**Total dryer energy requirement**	**327.46**	**MJ/day**

Energy for drying commercial PBAT is calculated by energy required for heating and evaporating the water and heating PBAT up to the reaction temperature, considering information in ***Table S3***. Additionally, for designing the dryer using hot air, the data in ***Table S4*** was used.

Table S4. *Parameters for designing the dryer.*

Parameter	Value	Unit
**Wet moisture % (X_i)**	2	%
**Dried moisture % (Xo)**	0	%
**Air moisture in (Y_i)**	1.5	%
**Air moisture out (Yo)**	3.2	%
**$T_{PBAT-in}$**	25	°C
**$T_{PBAT-out}$**	120	°C
**T_{air-in}**	120	°C
**$T_{air-out}$**	60	°C
**$h_{PBAT-in}$**	39.64	kJ/kg °C
**$h_{PBAT-out}$**	180.05	kJ/kg °C
**h_{air-in}**	264.11	kJ/kg °C
**$h_{air-out}$**	144.80	kJ/kg °C
**V^h_{air-in}**	1.14	$m^3/kg_{\text{dry air}}$
**$V^h_{air-out}$**	0.922	$m^3/kg_{\text{dry air}}$
**V_{air}**	2	m/s
**Gas flux (G'_s)**	0.455	kg/m^2 s
**Heat coefficient (U)**	151.926	W/m^3 K
**Length (H) theoretical**	3.141	M
**Length (H) Actual**	1.98	M

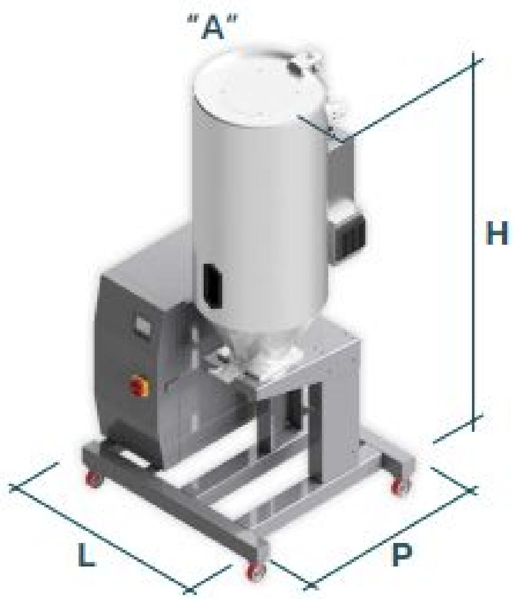

Figure S2. *DRYPLUS model DP160 desiccant wheel dryer for PBAT drying.*

Table S5. *Specification of industrial dryer.*

DRYPLUS DP160-H600-21 ft3	
DRYPLUS model DP160 desiccant wheel dryer	$12,352
648 lb drying hopper (400-liter, 14.1 ft3), 2.4" thick insulated drying hopper	$7,807
Mobile cart for DP160 dryer & 400-liter hopper	$1,494
**Total price**	**$21,653**

Reaction Calculation Method

A. Mass Balances: The mass values for each stream were reported in units of kg/day.

B. Reference state: the reference state for energy calculations was selected to be 25 °C and 1 bar pressure.

C. Higher Heating Values: The higher heating values (HHV) required for calculating the heat of reactions, were calculated using the Gaur and Reed formula [1].

$$HHV = (0.3491X_C) + (1.1783X_H) + (0.1005X_S) - (0.0151X_N) - (0.1034X_O)$$

Eq S1. *Gaur and Reed Expression for calculation of HHV*

(X_i = mass percentage and HHV is in MJ/kg)

D. Thermophysical Properties: The thermophysical properties like specific heat and latent heat of vaporization were extracted from the NIST database in Aspen or Polymer handbook [2].

E. Sample Calculation: A sample calculation is shown below for the energy balance around the PBAT-diol reactor where the following equation applies:

A. **PBAT-diol production reaction from PBAT and butanediol:**

$$[C_{22}H_{28}O_8]_{124} + 124\ HO-(CH_2)_4-OH \rightarrow 10.3\ [C_{26}H_{38}O_{10}]_{12}$$

$$\Delta \boldsymbol{H_{rxn}} = n_{PBAT-diol} \times \Delta H^f_{PBAT-diol} - n_{BDO} \times \Delta H^f_{BDO} - n_{PBAT} \times \Delta H^f_{PBAT}$$

$$= 10.3 \times (-374.579) - 124 \times (-505.3) - 1 \times (-2{,}401.925)$$

$$= -1{,}406.075\ \frac{MJ}{mol} \div 6{,}940\ \frac{g}{mol} \times 946.8\frac{kg}{day} \times \frac{1{,}000\ g}{kg} = -191.824\ \frac{MJ}{day}$$

For calculating the energy for PBAT-diol production from PBAT and butanediol, heat of formation of PBAT and butanediol are required. Additionally, for finding the heat of formation of PBAT and butanediol, heat of combustion of PBAT can be calculated and heat of formation of carbon dioxide and water be deducted. Same with butanediol formation, heat of carbon dioxide and water should be deducted from heat of combustion of butanediol.

A.1 PBAT formation:

$$2{,}728\ C + 1{,}736\ H_2 + 496\ O_2 \rightarrow [C_{22}H_{28}O_8]_{124}$$

$$\boldsymbol{\Delta H^f_{PBAT} = \Delta H^f_{CO2-H2O} - \Delta H^{comb}_{PBAT} = -2{,}401.925\ \frac{MJ}{mol}}$$

A.1.1 PBAT combustion:

$$[C_{22}H_{28}O_8]_{124} + 3{,}100\ O_2 \rightarrow 2{,}728\ CO_2 + 1{,}736\ H_2O$$

$$\Delta H^{comb}_{PBAT} = MW_{CPBAT} \times \Delta h^{comb}_{PBAT} = 31{,}230\ \frac{g}{mol} \times \frac{26.6\ KJ}{g} \div 1{,}000 = 832.202\ \frac{MJ}{mol}$$

A.1.2 Carbon dioxide and water formation:

$$2{,}728\ C + 3{,}596\ O_2 + 1{,}736\ H_2 \rightarrow 2{,}728\ CO_2 + 1{,}736\ H_2O$$

$$\Delta H^f_{CO2-H2O} = n_{CO2}\Delta H^f_{CO2} + n_{H2O}\Delta H^f_{H2O}$$

$$= 2{,}728\ mol \times (-393.52)\ \frac{KJ}{mol} + 1{,}736\ mol \times \frac{(-285.83)\frac{KJ}{mol}}{1{,}000}$$

$$= -1{,}569.723\ \frac{MJ}{mol}$$

A.2 PBAT-diol formation

$$312\,C + 228\,H_2 + 60\,O_2 \rightarrow [C_{26}H_{37}O_{10}]_{12}$$

$$\Delta \boldsymbol{H^f_{PBAT-diol}} = \Delta \boldsymbol{H^f_{CO2-H2O}} - \Delta \boldsymbol{H^{comb}_{PBAT-diol}} = \mathbf{-374.579}\ \frac{\boldsymbol{MJ}}{\boldsymbol{mol}}$$

A.2.1 PBAT-diol combustion:

$$[C_{26}H_{37}O_{10}]_{12} + 366\,O_2 \rightarrow 312\,CO_2 + 228\,H_2O$$

$$\Delta H^{comb}_{PBAT-diol} = MW_{BDO} \times \Delta h^{comb}_{PBAT-diol} = 6{,}940\ \frac{g}{mol} \times \frac{26.9\,KJ}{g} \div 1{,}000 = 186.631\,\frac{MJ}{mol}$$

A.2.2 Carbon dioxide and water formation:

$$312\,C + 426\,O_2 + 228\,H_2 \rightarrow 312\,CO_2 + 228\,H_2O$$

$$\Delta H^f_{CO2-H2O} = n_{CO2}\Delta H^f_{CO2} + n_{H2O}\Delta H^f_{H2O}$$

$$= 312\,mol \times (\,-393.52)\ \frac{KJ}{mol} + 228\,mol \times \frac{(\,-285.83)\ \frac{KJ}{mol}}{1{,}000}$$

$$= -187.947\ \frac{MJ}{mol}$$

While this reaction is exothermic, some energy input is required to move the reaction forward. This energy is estimated based on the energy requirement for the bench-top experiments, scaled up to industrial production capacity.

The energy from a hot plate goes partly to the reaction and partly to the environment through convection. Several studies have shown that the power was almost linearly related to the temperature below 600 K [3], [4]. Hence energy requirement for the samples at 200 °C with the maximum temperature of the instrument (200 °C) power demand is (200/200x100)% of the maximum power of the instrument. Specifications of the hot plate is listed in ***Table S6*** and the schematic of the hot plate is shown in ***Figure S3***.

Table S6. *Hot plate specifications*

**Corning® PC-420D**	
**Voltage**	120V/60 Hz
**Power**	698 Watts
**Maximum Operation Temperature**	200 °C

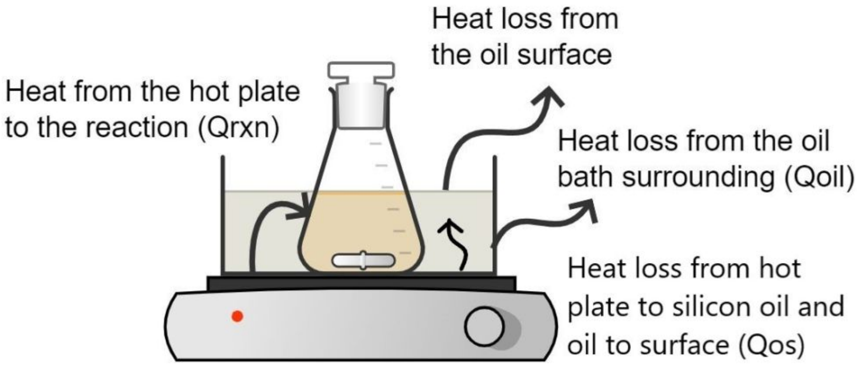

Figure S3. *Schematics of the hot plate and energy inputs and output.*

The maximum energy output of the hot plat working at 200 °C is as follows:

$$Q_p = 668\ \frac{J}{s} \times \frac{200}{200} \times 6\ h \times 3{,}600\ \frac{s}{h}\ \times \frac{kJ}{1{,}000\ J} = 15{,}076.8\ kJ$$

Energy loss to the surroundings from a 20 cm by 20 cm square hot plate and a 5 cm tall oil bath with a 15 cm diameter is as follows:

$$Q_{loss,1} = 2.5\ \frac{W}{m^2K} \times (182 - 25)\ K \times (3.14\ \times 0.15 \times 0.05)\ m^2 \times 6 \times 3{,}600\ s \times \frac{kJ}{1{,}000\ J} = 962.7\ kJ$$

$$Q_{loss,2} = 2.5\ \frac{W}{m^2K} \times (200 - 25)\ K \times \left(0.2\ \times 0.2 - 3.14 \times \frac{0.15 \times 0.15}{4}\right) m^2 \times 6 \times 3{,}600\ s \times \frac{kJ}{1{,}000\ J} = 289.6\ kJ$$

$$Q_{loss,3} = 2.5\ \frac{W}{m^2K} \times (182 - 25)\ K \times (0.15 \times 0.15)\ m^2 \times 6 \times 3{,}600\ s \times \frac{kJ}{1{,}000\ J} = 190.7\ kJ$$

$$Q_{loss,4} = 0.6\ \frac{W}{m^2K} \times (200 - 25)\ K \times \left(3.14 \times \frac{0.15 \times 0.15}{4}\right) m^2 \times 6 \times 3{,}600\ s \times \frac{kJ}{1{,}000\ J} = 40\ kJ$$

$$Q_{rxn} = (15{,}076.8 - 926.7 - 298.6 - 190.7 - 40) = 13{,}620\ kJ$$

This energy was required to produce 0.2 kg of PBAT-diol, for production of 946.8 kg/day of PBAT-diol we should scale up the numbers to:

$$Q_{rxn,PBAT-diol} = 13{,}620\ kJ\ \times \frac{1}{0.2\ kg} \times 946.8\ \frac{kg}{day} \times \frac{MJ}{1{,}000\ kJ} = 64{,}477\ MJ$$

This energy is much higher than the energy requirement for an industrial setting due to the large heat requirement for glassware with no insulation while an adiabatic reaction in an insulated reactor can be used. Herein, in our base case scenario, we have considered the energy from the exothermic is not used

elsewhere and in our sensitivity analysis, we assumed the maximum energy requirement is the scale up from the bench-top experiment is demanded.

All other energy input to the reactors is calculated in the same way.

Table S 7. *Summary of reactions and energy requirement*

Reaction	Heat of reaction (MJ/day)	Energy from scale-up (MJ/day)	Reaction duration (min)	Reaction temp. (°C)
**PBAT-diol production**	-191,824	64,477	360	200
**CPBAT production**	2,715	5,015	30	170
**CPBAT emulsification**	-3,555	2,820	45	77
**Starch dissolution**	--	6,607	45	90

The energy requirement for PBAT-diol production is the highest as the energy loss correlates with the temperature and duration of the reaction in bench-top experiments. Similarly, the energy requirement for the reaction with lower temperature, e.g. CPBAT emulsification at 77 °C is much lower.

To compare how much this method of energy calculation can be higher than the industrial scale, a study was found for calculating the breaking of the ester bonds in PETG. In this investigation, the energy for 1,000 kg PETG cards containing 920 kg PETG reaction with EG is estimated to be 800 MJ. Considering 18.57 wt% of PETG is the CHDM molecule, the rest has a similar structure to PET and the number of ester bonds should be the same. Hence, the mass of PET is 749.16 kg which equals 3,824.5 mol PET. In each repeating unit of PET, there are two ester bonds, therefore, there are 7,648 mol ester bonds present. It is assumed that 49.16/1,000 of the total energy for the reaction goes to breaking the ester bond and the energy for breaking one ester bond is as follows:

$$m_{PET} = 920 \times (1 - 0.1857) = 749.16\ kg$$

$$n_{PET} = 749.16\ kg \times \frac{1{,}000\ g}{1\ kg} \times \frac{1 mol}{192 g} = 3{,}910.87\ mol$$

$$E_{ester-PET} = \frac{800\ MJ}{3{,}910.87\ mol} = 0.2046\ \frac{MJ}{mol}$$

The number of ester bonds cleaved in reaction of PBAT with BDO can be estimated by knowing the mols of BDO. With 38 kg BDO which equals 422 mol and each PBAT monomer having three ester bonds, the energy required for PBAT-diol production can be estimated as follows:

$$n_{BDO} = \frac{38\ kg}{day} \times \frac{1{,}000\ g}{1\ kg} \times \frac{1\ mol}{92.12\ g} = 422\ \frac{mol}{day}$$

$$n_{eater-PBAT} = 422 \times 3 = 1{,}266\ \frac{mol}{day}$$

$$E_{PBAT-diol} = \frac{0.2046\ MJ}{mol} \times 1{,}266\ \frac{mol}{day} = 259.02\ \frac{MJ}{day}$$

Energy for rection comes from burning natural gas in a furnace. Composition of natural gas is up to 97% methane [5], hence it is assumed that it is only composed of methane. The furnace was designed in HYSYS Aspen software with the following information.

$$1CH_4 + 2(O_2 + 3.76N_2) \rightarrow CO_2 + 2H_2O + 7.52\,N_2$$

$$\Delta H = -393.5 + 2 \times (-285.83) - (-74.6) = -890.57\ \frac{kJ}{mol} \times \frac{1\ mol}{16\ g} \times \frac{1\ MJ}{1{,}000\ kJ} = -55.66\ \frac{MJ}{kg}$$

When the total amount of energy from scale up of the bench-top experiment is found, for the furnace designed in HYSYS Aspen, the natural gas enters the furnace at 200 °C with a flow rate of 12.5 kg/h while air has flowed to the furnace at the rate of 225.3 kg/h at 25 °C. The flare gas leaves the furnace at 505.5 °C. In this situation, the shell's overall volume is 9.7 m^3.

Reactor Design

For designing the reactor, 1st order reaction is assumed for all reactions. The graph below shows the reaction rate for PBAT-diol production that is 1st order with respect to PBAT concentration. For converting mol of PABT to concentration, melt density of PBAT and density of butanediol were used from Perry's handbook [6].

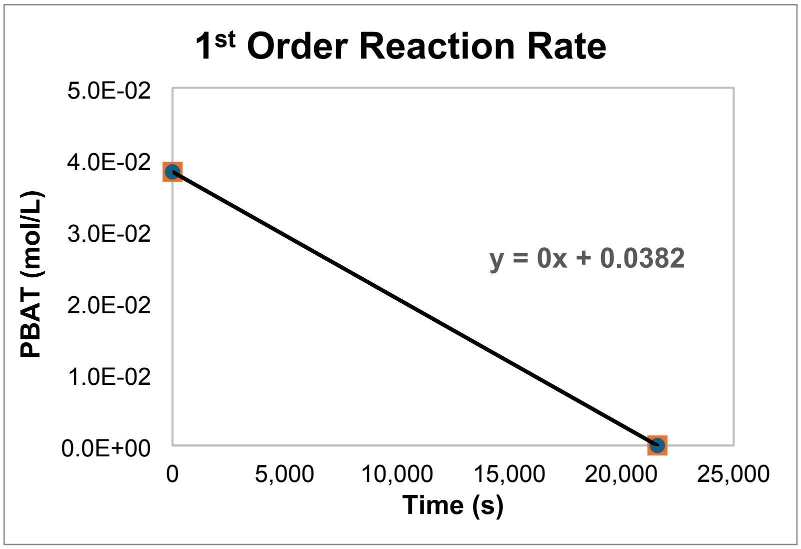

Figure S4. *The reaction rate for PBAT-diol production from PABT and butanediol*

$$n_{PBAT} = \frac{924.1\ kg/day}{36{,}230\ \frac{g}{mol} \div 1{,}000\ g/kg} = 29.59\ mol/day$$

$$n_{PBAT} = \frac{22.7\ kg/day}{90.12\ g/mol \div 1{,}000 g/kg} = 251.74\ mol/day$$

$$V_{tot} = \frac{924.1\ kg/day}{1.23\ kg/L} + \frac{22.7\ kg/day}{1.02\ kg/L} = 773.61\ L/day$$

$$C_{PBAT,0} = \frac{29.59\ mol/day}{773.61} = 0.038\ mol/L$$

$$V_{rxn,\ t} = \frac{C_{PBAT} V_{tot}}{-r} = \frac{0.038\ \frac{mol}{L} \times 773.61\ \frac{L}{day}}{-\left(-1.77 \times 10^{-6}\right)\ \frac{mol}{L\ s} \times \frac{3{,}600\ s}{h} \times \frac{24\ h}{day}} = 190.4\ L$$

Assuming that only 2-3rd of the reactor is filled, the actual volume would be:

$$V_{rxn,t} = \frac{3}{2} \times 190.4 = 290\ L$$

The cost of the reactor is found in ***Figure 13-15*** of the book *Plant Design and Economics for Chemical Engineers* [7].

Coating

CPBAT and/or starch are applied using a press coating machine. The oven is integrated into the coating machine. However, the evaporation of solvent is designed separately to give some insights about the impact of coating alone as well as drying.

Table S8. *Specifications of the coating machine*

Company	Coating machine type	Equipment price ($)	Power (kW)	Coating width (cm)	Temp (°C)	coating max speed (m/min)
Xiamen Simy Equipment	Customizable Coating Line Solvent Coater Machine with Oven	17,500	30	55	180	5

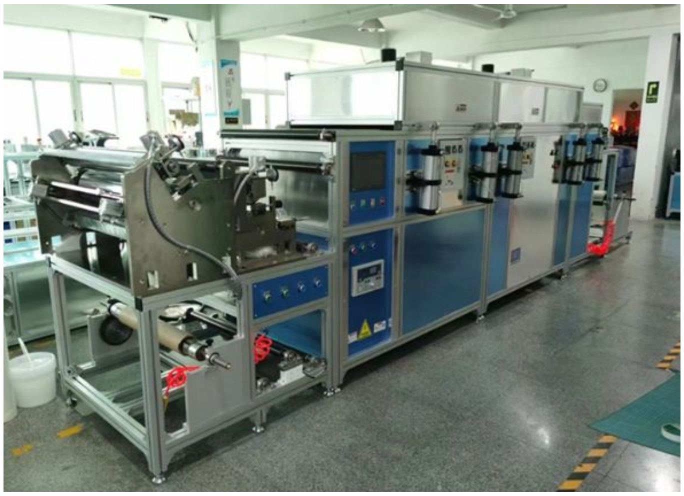

Figure S5. *Xiamen Simy Customizable Coating Line Solvent Coater Machine with Oven*

Since the maximum capacity of coating machine is limited to 7,200 meter per day, 5 coating machines can work with lower coating speed than 5 m/min to coat 1,000 kg/day of CPBAT. Coating starch is analogous, and 5 coating machines should work with lower speed to deliver the required starch coated papers. Data required for energy calculation and price of the coating machine is summarized in **Table S9** and **Table S10**.

Table S9. *Data for CPBAT coated unbleached Kraft paper from ULINE (75 lb)*

Parameter	Value	Unit
Paper basis weight	0.122	kg/m^2
Coating weight/area	0.060	kg/m^2
Total weight/area	0.182	kg/m^2
Coating area	16,694.5	m^2
Length of the coated paper	29,811.6	M
Max length per day	7,200	m
Number of coating machine	5	NA
Energy for coating CPBAT	53,660.9	MJ/day

Table S10. Data for starch coated on unbleached Kraft paper from ULINE (75 lb)

Parameter	Value	Unit
Starch weight per area	0.017	kg/m^2
Total starch weight	278.8	kg
Coating area	16,694.5	m^2
Length of the coated paper	29,811.6	m
Max length per day	7,200	m
Number of coating machine	5	NA
Energy for coating CPBAT	47,435.0	MJ/day

Data for ULINE 50 lb and 75 lb Kraft paper as well 50 lb ULINE Poly coated paper (polyethylene coated paper) was available in their website [5]. Since in our experiments, 50 lb Kraft paper was used, data for 75 lb Poly coated paper was estimated based on 75 lb Kraft paper and 50 lb Poly coated paper. **Table S11** is the summary of specifications of each paper as well as the price per area.

Table S11. Specification and price of Kraft paper and Poly coated paper (50 lb and 75 lb) from Uline.

Poly coated paper type	ULINE Kraft paper (50 lb)	ULINE Kraft paper (75 lb)	ULINE Poly coated paper (50 lb)	ULINE Poly coated paper (75 lb)
Basis weight (kg)	50	75	50	50
Length (m)	219.50	144.80	182.90	182.90
Width (cm)	56	56	56	56
Area (m^2)	122.90	81.08	102.41	81.08
Weight per roll	0.08	0.12	0.10	0.15
Price ($/$m^2$)	0.28	0.41	0.64	0.94

Drying coated paper

As mentioned earlier, while the drying step is integrated into the coating machine, energy for heating and evaporation of solvents were estimated separately and deducted from the coating process to have a clearer idea of how much energy is allocated to coating versus drying. Energy for evaporating water-ammonia mixture was calculated by modeling the process in HYSYS Aspen software with the inputs and outputs derived from the model summarized in **Table S12**.

Table S12. *Water-ammonia evaporation modeling using HYSYS Aspen*

Parameter	Inlet	outlet	unit
Vapor fraction	0	1	NA
Flow rate	2,615.4	2,615.4	kg/day
Ammonia mass fraction	0.07	0.07	NA
Water mass fraction	0.93	0.93	NA
Temperature	50	180	°C
Pressure	100	1071	kPa
Molar enthalpy	-2.69E+05	-2.23E+05	kJ/kmol
Duty		**6,753.82**	**MJ/day**

Table S13. *Summary of equipment costs and sizing for CPBAT, CPABT-K and CPBAT-S production.*

Equipment item	Size (m^3)	Equip. Cost ($)	Installed Equip. Cost (CPBAT) ($)	Installed Equip. Cost (CPBAT-K) ($)	Installed Equip. Cost (CPBAT-S) ($)	Ref.
**Rotary dryer I**	1.32	108,265		162,398	162,398	[8], [9]
CSTR (PBAT-diol production)	0.29	24,228	162,398	24,228	24,228	This study, [7]
Filter	NA	3,634	24,228	3,634	3,634	[10]
CSTR (CPBAT production)	*0.03*	12,114	3,634	23,985	23,985	This study, [7]
CSTR (CPBAT emulsification)	0.16	18,171	3,162	18,171	18,171	This study, [7]
Furnace I	9.70		18,171			This study, [7]
Isolated storage tank (CPBAT)	0.3	10,095	*NA*	10,095	10,095	This study, [7]
Coating machine (CPBAT)	NA	87,500	NA	135,625	135,625	[11]
CSTR (starch dissolution)	*0.68*	36,341.25	NA	NA	71,956	This study, [7]
**Furnace II**	NA		NA	NA		This study, [7]
Isolated storage tank (starch)	8	16,151.67		NA	31,173	This study, [7]
**Coating machine for starch**	NA	108,265	NA	NA	162,398	[11]

Sensitivity analysis

Ionization solvent condensation

The mixture of ammonia and water is not recovered in the base case scenario, however, the impact of partial recovery on the cost is studied in the sensitivity analysis. In the base case scenario, it is assumed that 60% of the solvent is lost during the drying step, and 40% was recovered. By changing the recovery rate to 20% and 60%, the sensitivity of the minimum selling price to the solvent recovery was estimated. Energy savings for the condensation process and the cost of the condenser were found by modeling the process in HYSYS Aspen.

Table S14. *Water-ammonia condensation modeling using Aspen HYSYS (20% loss)*

Parameter	Inlet	outlet	unit
Vapor fraction	1	0	NA
Flow rate	2,092.3	2,092.3	kg/day
Ammonia mass fraction	0.07	0.07	NA
Water mass fraction	0.93	0.93	NA
Temperature	180	25	°C
Pressure	1,000	100	kPa
Molar enthalpy	-2.23E+05	-2.72E+05	kJ/kmol
Duty (20% material loss)	**-5,236.80**		**MJ**

Heat of reaction

The heat of the reaction for PBAT-diol production and CPBAT dissolution is negative, because of the exothermic nature of the reaction. However, as mentioned earlier, the temperature at which the PBAT-diol production is happening is high and it is very probable that in the real world some heat might be required to move the reaction forward. This reaction is analogous to ester bond breakage in PET/PETG. One analysis of PETG depolymerization in a pilot plant scale has shown that to break the ester bonds in ~912 kg PETG and convert it to BHET, 800 MJ energy is required [12]. Herein, to analyze how sensitive the price is to the energy requirement for the reactions, we have scaled up the energy from bench-top scale to industrial scale for both exothermic reactions. We keep in mind that in bench-top experiments most of the heat coming from hot plates is lost to the environment as the reaction is open air and the apparatus is glass. To keep this assumption consistent, the overall heat of the reaction is estimated to be the sum of all three heats that are calculated from bench-top experiments. Then, 50% of this energy is used as input for

sensitivity analysis. This energy comes from burning natural gas in a furnace. Hence, for each energy demand, the design of the furnace as well as the fuel requirement is changed, hence the cost is changed.

Supporting Information

Bibliography

[1] T. B. Gaur, S. and Reed, "Thermal Data for Natural and Synthetic Fuels," *Technol. Eng.*, 1998.

[2] Z. Raheem, "POLYMER DATA," no. June, 2019.

[3] J. Spannhake, O. Schulz, A. Helwig, A. Krenkow, G. Müller, and T. Doll, "High-temperature MEMS heater platforms: Long-term performance of metal and semiconductor heater materials," *Sensors*, vol. 6, no. 4, pp. 405–419, 2006.

[4] S. Santra *et al.*, "Post-CMOS wafer level growth of carbon nanotubes for low-cost microsensors - A proof of concept," *Nanotechnology*, vol. 21, no. 48, 2010.

[5] "Uline Kraft paper," *2024*. [Online]. Available: https://www.uline.com/Product/Detail/S-1311/Kraft-Paper-Wrap/50-lb-Kraft-Paper-Roll-24-x-720?pricode=WB0421&gadtype=pla&id=S-1311&gad_source=1&gclid=Cj0KCQjwwMqvBhCtARIsAIXsZpYRbOKRULoYgu4FpdlTtUCXqiLXEd4ZNwxNdbEUUJE-ArqYRLhb84gaAgxHEALw_wcB.

[6] "Perry's Chemical Engineers' Handbook."

[7] M. S. Peters and K. D. Timmerhaus, *Plant design and economics for chemical engineers*. Journal of Chemical Education, 1958.

[8] "Summit Systems DRYPLUS Rotary Dryer, model DP160 desiccant wheel dryer," 2023. .

[9] "Rotary Dryer Heat and Mass Balance Calculation," *2023*. [Online]. Available: https://powderprocess.net/Spray_Drying/Spray_Drying_Heat_Mass_Balance.html.

[10] A. Singh *et al.*, "Techno-economic, life-cycle, and socioeconomic impact analysis of enzymatic recycling of poly(ethylene terephthalate)," *Joule*, vol. 5, no. 9, pp. 2479–2503, 2021.

[11] "Xiamen Simy Equipment Customizable Coating Line Solvent Coater Machine with Oven," 2023. [Online]. Available: https://www.alibaba.com/product-detail/Customizable-Coating-Line-Solvent-Coater-Machine_1600795313902.html?spm=a2700.galleryofferlist.p_offer.d_title.17ca5608lDiVNu&s=p.

[12] M. Larrain *et al.*, "Techno-economic assessment of mechanical recycling of challenging post-consumer plastic packaging waste," *Resour. Conserv. Recycl.*, vol. 170, no. April, p. 105607, 2021.
